# Outcomes of trauma education workshop in Vietnam: improving diagnostic and surgical skills

**DOI:** 10.1186/s12909-020-02195-1

**Published:** 2020-08-15

**Authors:** Sugy Choi, Jieun Kim, Jongho Heo, Dung Thi Ngoc Nguyen, Son Hong Nguyen, Woong-Han Kim

**Affiliations:** 1 Department of Health Law, Policy & Management, Boston University School of Public Health, Boston, MA USA; 2 JW LEE Center for Global Medicine, Seoul National University College of Medicine, Seoul, Republic of Korea; 3 National Assembly Futures Institute, Seoul, Republic of Korea; 4 Military Hospital 175, Ho Chi Minh City, Vietnam; 5 Department of Thoracic and Cardiovascular Surgery, Seoul National University College of Medicine, Seoul, Republic of Korea

**Keywords:** Trauma education, Global health, Program evaluation

## Abstract

**Background:**

Unintentional injuries have emerged as a significant public health issue in low- and middle-income countries (LMIC), especially in Vietnam, where there is a poor quality of care for trauma. A scarcity of formal and informal training opportunities contributes to a lack of structure for treating trauma in Vietnam. A collaborative trauma education project by the JW LEE Center for Global Medicine in South Korea and the Military Hospital 175 in Vietnam was implemented to enhance trauma care capacity among medical staff across Ho Chi Minh City in 2018. We aimed to evaluate a part of the trauma education project, a one-day workshop that targeted improving diagnostic and surgical skills among the medical staff (physicians and nurses).

**Methods:**

A one-day workshop was offered to medical staff across Ho Chi Minh City, Vietnam in 2018. The workshop was implemented to enhance the trauma care knowledge of providers and to provide practical and applicable diagnostic and surgical skills. To evaluate the workshop outcomes, we utilized a mixed-methods survey data. All participants (*n* = 27) voluntarily completed the post-workshop questionnaire. Quality of contents, satisfaction with teaching skills, and perceived benefit were used as outcomes of the workshop, measured by 5-point Likert scales (score: 1–5). Descriptive statistics were performed, and open-ended questions were analyzed by recurring themes.

**Results:**

The results from the post-workshop questionnaire demonstrated that the participants were highly satisfied with the quality of the workshop contents (mean = 4.32 standard deviation (SD) = 0.62). The mean score of the satisfaction regarding the teaching skills was 4.19 (SD = 0.61). The mean score of the perceived benefit from the workshop was 4.17 (SD = 0.63). The open-ended questions revealed that the program improved their knowledge in complex orthopedic surgeries neglected prior to training.

**Conclusions:**

Positive learning experiences highlighted the need for the continuation of the international collaboration of skill development and capacity building for trauma care in Vietnam and other LMIC.

## Background

Unintentional injuries, including traffic accidents, have emerged as a leading cause of death and disability representing a significant public health issue in low- and middle-income countries (LMIC), especially in Vietnam [1]. Over 9000 deaths, 24,000 injuries, and 25,000 traffic accidents have been reported in 2014 in Vietnam [2]. The estimated traffic accident deaths have increased from 24.5 per 100,000 in 2013 to 26.4 per 100,000 in 2016, a much higher rate than in neighboring countries, such as Laos (16.6 per 100,000) or Cambodia (17.8 per 100,000) [3]. Due to its rapid urbanization, Ho Chi Minh City has the highest number of traffic accident deaths in Vietnam; from 2013 to 2015, about 2500 traffic accident deaths have occurred [4, 5].

Despite the high burden of traffic accidents, the quality of care for trauma has been a continuing challenge in Ho Chi Minh City and Vietnam overall [1]. In Ho Chi Minh City, in order to address the traffic accident morbidity and mortality rates, the Military Hospital 175 in 2015 received $2.200 billion Vietnam Dong (95 million USD) to establish a trauma center. The construction of the trauma hospital building was completed in December 2019. Yet, Vietnam does not have an established trauma specialty surgeon program [6]. A scarcity of formal and informal training opportunities contributes to this lack of a system for treating trauma.

The collaboration between the Military Hospital 175 and Seoul Medical University College of Medicine officially began with a memorandum of understanding (MoU) in 2013. Responding to the plan to establish a new Trauma and Orthopedics Center within Military Hospital 175 in 2015, the JW LEE Center for Global Medicine, Seoul National University College of Medicine in the Republic of Korea implemented a project for strengthening trauma care capacity with the Military Hospital 175. Training is one of the widely delivered intervention methods to increase trauma care capacity in LMICs [7]. Three-month invitational medical training fellowship programs (2016–2018) and a one-day workshop in Ho Chi Minh City were offered (2018). The main goal of the overall medical education project was to provide continuing education for clinicians to improve trauma care. After conducting a needs assessment of the trauma care team who participated in the invitational training program in Korea, faculty from Korea formed the content of the workshop including microsurgery and reconstruction, injuries and fractures, and trauma intensive care unit management.

The objective of the workshop was to train participants in trauma-care knowledge and skills and to apply their newly gained skillsets. In this paper, we seek to describe the structure of the overall project for strengthening trauma care capacity, to review the workshop contents, and to discuss participants’ initial responses to the workshop in order to improve future workshops and training.

## Methods

We used a quantitative-dominant mixed-methods research design to evaluate the trauma evaluation project.

### Study site

The Military Hospital 175, a tertiary hospital that operates under the Vietnamese National Ministry of Defense in Ho Chi Minh City, is a central military hospital in the Southern region of Vietnam that carries out medical services for senior officers in the army, but it is also open to the public. It is a teaching hospital that educates and trains clinicians including surgeons, orthopedists, internists, and residents.

### Sample

There were a total of 111 healthcare professionals (physicians, nurses, and administrators) employed in the target hospital departments (Trauma and Orthopedic, Plastic and Reconstructive Surgery, Spinal Surgery, General Surgery, Burn Surgery, Emergency Medicine) at the time of study recruitment, and 63 (physicians and nurses) were available and registered to participate in the study. Recruiting internal medical staff was prioritized and the primary method of recruitment was by invitation via e-mail and phone calls. Additionally, since the Military Hospital 175 is a public teaching hospital, the communication department disseminated newsletters and dispatch (for military institutions) to the hospitals in the Ho Chi Minh City area for recruitment as well. Of 74 total registrants, 27 (36%) attended the workshop and were asked to complete the post-workshop questionnaire. About 40% of the attendants came from another institution (*n* = 11). A total of 27 provider participants (physicians and nurses) voluntarily completed the questionnaire and 25 participants completed all of the questions. We obtained a 100% response rate.

### Intervention

Strategies used in the project were based on Kern’s framework for curriculum development, which describes a logical and systematic approach to developing, implementing, and evaluating medical education programs [8]*.* Drawing on the six steps (problem identification and needs assessment, targeted needs assessment, goals and objectives, educational strategies, implementation, and evaluation and feedback), the intervention sought to educate trauma professionals in Vietnam by conducting an education program emphasizing the local needs.

#### Problem identification and needs assessment

First, a general needs assessment was conducted based on the published literature and reports on the need for trauma care, and country-specific needs were identified.

#### Targeted needs assessment

A specific needs assessment was conducted with stakeholders (hospital leadership, surgeons, and physicians) at the Military Hospital 175. A stakeholder analysis was conducted at the 20th scientific conference hosted by the Military Hospital 175 in 2015. One of the trauma specialists attended the conference and met with several stakeholders to discuss the local problems and needs. Based on the stakeholder meetings, a need for essential resources was recognized along with a need for facility, equipment, and personnel. Skill-based training was prioritized for this partnership between the two institutions because the construction was a responsibility of the Vietnamese government. The discussions elucidated the need for and willingness to participate in the proposed training programs.

#### Goals and objectives

We used both needs assessments to develop the goals and objectives of the curriculum: 1. to share experiences and skills in the field and 2. to teach the concept of trauma patient care system in order to strengthen the capacity of trauma care at the Military Hospital 175.

#### Educational strategies

The Strengthening Trauma Care Capacity Project is a multimodal educational program. The first part of the project is the Trauma Care Capacity Strengthening Training Fellowship Program in Korea (2016–2018), which is a 3-month invitational training program. The scope of the training includes lectures, mentorships, hands-on learning, visual learning, and field trips to various trauma centers in Korea. The Military Hospital 175’s leadership selected trainees based on ability, English competency, and needs and provided training expenses for all three months except airfare. The coordinators interviewed trainees prior to their fellowship to understand the training needs, and the examples of their requested areas included Microsurgery, Traumatic Emergency, Knee Arthroscopy, Sports Medicine, and Hand Surgery. The second part of the project is the one-day Trauma Care Strengthening Workshop (2018). The Vietnamese physicians led the curriculum development because the trauma system between Vietnam and Korea is different. The Military Hospital 175’s leadership and surgeons discussed what program they envisioned and the detailed lecture topics via e-mails. Professors from Korea who instructed the trainees during the Trauma Care Capacity Strengthening Training Fellowship Program were first consulted to develop and teach the workshop. The content of the lecture was consulted with Vietnamese professors in charge.

#### Implementation

We built a collaborative partnership between Vietnamese and Korean health care professionals, administrators, and coordinators to implement the program. Table 1 shows the workshop’s contents and its duration. The main aims of the workshop were to enhance the knowledge of providers about trauma care (e.g. orthopedic care, microsurgery and reconstruction, injuries and fractures, trauma intensive care unit management) and to provide practical and applicable skills (i.e. diagnostic and surgical skills). There was no cost for clinicians to attend the workshop, and lunch was provided for all participants.

**Table 1:** Workshop session contents

Topic	Format	Duration
Orthopedic and Trauma Hospital 175	Forum	55 min
Characteristics of Fractures in Children	Didactic and Q&A session	30 min
	Discussion	
	Case Studies	
	Imagery	
	Providing cues	
	Quiz	
Pediatric Elbow fractur	Didactic and Q&A session	30 min
	Discussion	
	Case Studies	
	Imagery	
	Providing cues	
	Scenario-based risk info	
Microsurgery & Reconstruction for Trauma Patients	Didactic and Q&A session	60 min
	Discussion	
	Imagery	
	Providing cues	
Reconstruction for Facial Soft Tissue Injuries and Fractures	Didactic and Q&A session	60 min
	Discussion	
	Imagery	
	Providing cues	
	Scenario-based risk info	
Child Abuse	Didactic and Q&A session	30 min
	Case Studies	
	Changing trend in Korea	
Management of Trauma Center	Didactic and Q&A session	30 min
	Trial and errors	
	Recommendations for future	
Trauma Care for Critical Patients: Seamless Care from the Scene to TICU	Didactic and Q&A session	60 min
	Discussion	
	Case Studies	
	Imagery	
	Providing cues	
	Statistical reviews	

#### Evaluation and feedback

We developed an evaluation plan for the program including the workshop. The evaluation questionnaire was distributed at the end of the workshops to assess the quality of the didactics. The participants were asked to fill out the form and they submitted it immediately after the workshop.

### Data collection

The program coordinators conducted pilot tests of the questionnaire and made appropriate modifications to ensure readability and understanding. The surveys were translated from English to Vietnamese. Institutional Review Boards at the Seoul National University Hospital approved the research including the protocol and instruments (#1910–064-1070).

### Measures

A 14 item questionnaire using a 5-point Likert scale (1 = strongly disagree, 2 = disagree, 3 = neutral, 4 = agree, 5 = strongly agree) was used to measure participant satisfaction with the workshop and its contents. The quality of contents, satisfaction with teaching skills and tools, and perceived benefits were used as outcomes of the workshop. The participants rated the overall quality of education, whether the teaching methods used helped them to learn effectively, whether course handouts and texts reinforced their learning, whether the course objectives were met, and whether the course satisfied their own needs and expectations. Open-ended questions were asked to explore detailed information, such as their satisfaction and experiences after the training, and to obtain recommendations for future workshops. The questionnaire also asked for participants’ demographic information: name, gender, age, title, specialty, years of experience, and employment at the Military Hospital 175.

### Analysis

All of the participants completed the survey (*n* = 27), and 25 participants fully completed the survey. Two participants did not complete the evaluation part of the survey; therefore, they were excluded from the evaluation part of the analysis. Mean item descriptive statistics were calculated and all quantitative data were analyzed using STATA software version 13. For qualitative data, first, two researchers read all of the answers and independently generated lists of initial themes. The team met to discuss the themes into a set of codes. We coded open-ended answers to extrapolate recurring statements and themes. One researcher coded the transcripts using excel. Coded transcripts were qualitatively analyzed, and two senior researchers reviewed the codes. All authors agreed with the final themes.

## Results

Table 2 summarizes the demographic characteristics of the participants. A total of 27 respondents completed the post-evaluation questionnaire (19 doctors, 2 nurses, and 6 rehabilitation therapists). Most of the respondents were employed at the Military Hospital 175 (74%), and many of them indicated that trauma and orthopedic was their specialty (60%). On average, most of the respondents had been working for 8 years in their respective specialties. The mean age of the respondents was 35 years old. Most of the respondents were male (78%).

**Table 2:** Demographic characteristics of the participants (*n* = 27)

Surveyed Characteristics	n (%)
**Title**	
Doctor	19 (70.4)
Nurse	2 (7.4)
Rehabilitation therapist	6 (22.2)
**Specialty**	
Trauma and Orthopedic	16 (59.3)
Neurosurgery	1 (3.7)
Rehabilitation	7 (25.9)
General Surgery	3 (11.1)
**Mean years of experience in the specialty (sd)**	8 (7.9)
**Gender**	
Male	21 (77.8)
Female	6 (22.2)
**Mean age (sd)**	35 (9.7)
**Employed at the Military Hospital 175**	20 (74.1)

The overall evaluation of the workshop, given by the respondents, is summarized in Table 3. They agreed that the overall quality of the workshop contents was satisfactory (mean = 4.32 standard deviation (SD) = 0.62). They were also satisfied with the use of course handouts, and texts reinforced their learning (mean = 4.19 (SD = 0.63)). The mean score of satisfaction with the teaching skills was 4.28 (SD = 0.58). The mean score of the perceived benefit of the workshop was 4.17 (SD = 0.63).

**Table 3:** Evaluation of the didactics using Likert scale (5 = Strongly Agree, 4 = Agree, 3 = Neutral, 2 = Disagree, 1 = Strongly Disagree) (*n* = 25)

Evaluation of the Workshop	Summary mean scores (sd)
Overall quality of education was satisfactory	4.32 (0.62)
The teaching methods used helped me learn effectively	4.28 (0.58)
Course handouts & texts reinforced my learning	4.19 (0.63)
Course objectives were met	4.23 (0.56)
The course satisfied my own needs and expectations	4.17 (0.63)

The qualitative answers corroborated the quantitative results that the didactics were useful for the participants. The qualitative findings supported the quantitative results that the workshop improved participants’ knowledge of complex orthopedic surgeries, which had not been addressed before the training. The major strengths of the workshop were identified as the customized training approach that gauged teaching to the participant’s level of competency, based on findings from the needs assessment. Participants indicated that the content was practical and that they could immediately apply their learned skills.

## Discussion

The goal of this study was to evaluate the one-day trauma education workshop conducted in Vietnam, 2018. Provider participants had varying levels of experience with trauma, but all of the participants found the workshop to be useful. Participants were highly satisfied with the workshop. The satisfaction metrics were corroborated by answers that were presented for the open-ended questions. Participants indicated that the workshop enhanced clinical knowledge related to trauma care, especially fractures in children and management of the trauma center.

Increasing the knowledge of providers is an important factor for quality trauma care. Our study found that clinicians supported attending a one-day workshop in order to further their knowledge and skills. The findings support a recent study that demonstrated improvement in cognitive and surgical skills after participating in trauma courses [9]. The study reported improvement in staffing, increased equipment and training as well [9]. As the current project continues, in future observations, we also hope to measure the effects beyond individual participants. In addition, participants expressed interest in learning highly skilled procedures or technologically advanced procedures. Additional support, refresher courses, and ongoing mentorship are needed to sustain their skills.

There are several limitations to this study. The findings should be interpreted with caution due to the small size of the convenience sample. It may not be generalizable to all of the clinicians in the Ho Chi Minh City area. Only 36% of the registered providers attended the workshop. This may be due to the length of the workshop (full day) and their availability as the workshop was held on a weekday. All participants responded to the demographic questions, and 93% of the participants fully answered both demographic and evaluation questions, which was higher than expected. The majority of the participants were from the Military Hospital 175, reflecting our target population. However, we observed that study participation was uneven across specialty and across the profession. This may be due to the scheduling conflicts due to the long length of the workshop. For future workshops, we can identify ways to improve attendance diversity by ensuring stakeholder buy-in from all appropriate specialties. Another limitation is the fact that the workshop was a one-time event, making the study cross-sectional instead of longitudinal. In terms of evaluation design considerations, although randomization may be a possibility, this would likely be more expensive and less manageable. More work is needed to track longitudinal outcomes for methodologically rigorous intervention and evaluation. Although the sample size is small, this type of study can be replicated in other cities, and lessons learned can be applied to other settings in LMIC that are implementing trauma care education programs. The post-workshop questionnaire did not objectively test the post knowledge of the participants, a major limitation. This study, therefore, only examines self-reported participant reaction to the workshop, the minimal level of training effectiveness. Future workshops will include pre-tests and post-tests to measure changes in the participants’ knowledge and observations. Although using a 5-point Likert scale is widely accepted, the reliability and validity of the questionnaire need to be further tested for future evaluations.

## Conclusion

In order to address the high morbidity and mortality from traffic accidents in Ho Chi Minh City, a trauma care workshop was developed by Military Hospital 175 in collaboration with the JW LEE Center for Global Medicine and Seoul National University. Participants found the major strengths of the workshop as teaching relevant and practical knowledge. The new Trauma and Orthopedics Center of Military Hospital 175 was completed in late 2019, and positive learning experiences highlighted the need for the continuation of the international collaboration of capacity building for trauma care in Ho Chi Minh City. Trauma care improvement heavily relies on the regional, organizational, and provider and patient-level factors. Future collaborations should not only focus on developing the skills of providers but also on engaging and educating patients and other relevant stakeholders with the goal of reducing traffic accident mortality.

## Data Availability

The datasets used and/or analyzed during the current study are available from the corresponding author on reasonable request.

## Appendix

**Table 4:** Evaluation of the individual didactics using Likert scale (5 = Strongly Agree, 4 = Agree, 3 = Neutral, 2 = Disagree, 1 = Strongly Disagree) (*n* = 25)

Evaluation of the Didactic	Summary mean scores (sd)
Characteristics of Fractures in Children	4.23 (0.55)
Pediatric Elbow fracture	4.23 (0.56)
Microsurgery & Reconstruction for Trauma Patients	4.30 (0.66)
Reconstruction for Facial Soft Tissue Injuries and Fractures	4.26 (0.57)
Child Abuse	4.26 (0.56)
Management of Trauma Center	4.13 (1.0)
Trauma Care for Critical Patients: Seamless Care from the Scene to TICU	4.26 (0.59)

## Abbreviation

LMIC: Low- and middle-income countries
